# Association of Interleukin-6 and Interleukin-10 Genotypes With Radiographic Damage in Rheumatoid Arthritis Is Dependent on Autoantibody Status

**DOI:** 10.1002/art.22814

**Published:** 2007-08

**Authors:** I Marinou, J Healy, D Mewar, D J Moore, M C Dickson, M H Binks, D S Montgomery, K Walters, A G Wilson

**Affiliations:** 1 I. Marinou, BSc, J. Healy, BSc, D. Mewar, MB, PhD, MRCP, D. J. Moore, MB, FRCR, K. Walters, PhD, A. G. Wilson, MB, PhD, FRCP: University of Sheffield, Royal Hallamshire HospitalSheffield, UK; 2 M. C. Dickson, PhD, M. H. Binks, MB, MRCP, D. S. Montgomery, PhD: GlaxoSmithKline R&DStevenage, UK

## Abstract

**Objective:**

Recent evidence has highlighted a major genetic contribution to radiographic damage in rheumatoid arthritis (RA). The objective of this study was to determine whether genetic variants in the loci for interleukin-1 (IL-1), IL-6, IL-10, protein tyrosine phosphatase N22 (PTPN22), and selenoprotein S are associated with radiographic damage.

**Methods:**

Modified Larsen scores of radiographic damage were determined in a cross-sectional population of patients with RA (n = 964). Rheumatoid factor (RF) and anti–cyclic citrullinated peptide (anti-CCP) were also assayed. The Kruskal-Wallis nonparametric test was used to compare median radiographic damage scores across genotype groups, followed by the Cuzick nonparametric test for trend to assess gene-dose effects.

**Results:**

An allele-dose association of IL-6 −174G with increasing radiographic damage was present (*P* = 0.005), but only in patients who were RF positive (*P* = 0.004) or anti-CCP positive (*P* = 0.01). Patients with the IL-10 −592CC genotype had more extensive radiographic damage than did those with the AC or AA genotype (*P* = 0.006), but this was observed only among patients who were RF negative (*P* = 0.002) or anti-CCP negative (*P* = 0.002). However, RF status and anti-CCP status were not associated with the IL-6 or IL-10 genotype. No other genetic associations were detected, apart from a marginal association of PTPN22 +1858T with increased radiographic damage.

**Conclusion:**

The reported associations of IL-6 −174G with high IL-6 production and IL-10 −592 with low IL-10 production and our own results support a role of genetically determined dysregulated cytokine production in disease severity. The lack of association of these genotypes with RF and anti-CCP antibody status suggests that they act downstream of autoantibody production. We conclude that IL-6 and IL-10 genotypes may be useful in predicting disease severity in autoantibody-positive and autoantibody-negative patients, respectively.

Rheumatoid arthritis (RA) is the most common autoimmune inflammatory joint disease, with a prevalence of 1%. The etiology of RA is multifactorial and includes a significant genetic component, with a relative risk of recurrence of 5–10 in siblings of probands (1). Many genetic linkage and association studies have implicated a group of alleles of the DRB1 gene encoding a similar sequence, termed the shared epitope (2,3). Recent evidence has also implicated the common allele of the R620W single-nucleotide polymorphism (SNP) (rs2476601) in the hematopoietic-specific protein tyrosine kinase, protein tyrosine phosphatase N22 (PTPN22), with susceptibility to several autoimmune diseases, including RA (4).

The severity of RA varies from mild self-limiting arthritis to an aggressive systemic disease associated with a significantly reduced life expectancy. Several validated systems for scoring radiographic damage are widely used to assess disease progression or severity, including the Sharp and Larsen scores (5,6). More severe radiographic damage correlates with the presence of rheumatoid factor (RF) and anti–cyclic citrullinated peptide (anti-CCP) antibodies, with the presence of both being associated with more severe changes compared with the presence of either of the biomarkers alone (7). Longitudinal studies have revealed a strong correlation of total inflammation load, as assessed by C-reactive protein (CRP) levels over time, with greater radiographic damage (8). A recent report described the usefulness of measurement, at the time of disease presentation, of a range of biomarkers, including RF, anti-CCP antibodies, DRB1 alleles, and CRP and suggested that these variables could be used to predict damage to the hand and foot joints; however, the variance of radiographic joint damage that could be explained by these measurements was only 44% at 5 years and only 32% at 10 years, suggesting that other variables contribute to joint damage (9).

Relatively few studies have addressed the role of genetics in relation to radiographic joint damage. A small study compared the radiographic progression in monozygotic twins, dizygotic twins, and unrelated pairs of patients and found that variation was maximal in unrelated patients, followed by dizygotic twins, and was least in monozygotic twins, suggesting genetic input (10). A relationship between DRB1 alleles and disease severity has been reported, but recent evidence suggests that it is secondary to production of anti-CCP antibodies (11), particularly in RF-negative patients (5). Many studies have shown genetic associations with RA severity; however, these studies used different criteria to measure disease severity, such as the presence of rheumatoid nodules, a history of joint replacement surgery, earlier age at disease onset, or radiographic scoring methods. Furthermore, they mostly involved relatively small numbers of patients and did not assess the relationship with established biomarkers such as autoantibodies (12).

A central feature of RA is a relative imbalance of cytokine production, with a relative excess of proinflammatory molecules including interleukin-1 (IL-1), IL-6, and tumor necrosis factor (TNF) compared with antiinflammatory mediators such as IL-10 (13). The successful therapeutic use of inhibitors of proinflammatory cytokines underlines the importance of these molecules in driving rheumatoid inflammation and tissue damage. The production of these molecules is controlled at the levels of transcription, messenger RNA stability, and translational efficiency. Recently, selenoprotein S (SEPS) was identified as an important regulator of IL-1, TNF, and IL-6, with plasma levels of these proteins correlating with functional promoter single-nucleotide polymorphisms (SNPs) in SEPS (14).

In this genetic study, we examined whether SNPs in IL-1, IL-6, IL-10, PTPN22, and SEPS are associated with radiographic damage in a large cross-sectional cohort of patients with RA, and determined whether the associations detected were independent of production of RF and anti-CCP. Variants were chosen based on previous reports of functional relevance or associations with RA.

## PATIENTS AND METHODS

### Patients

Nine hundred sixty-four patients fulfilling the American College of Rheumatology (formerly, the American Rheumatism Association) criteria for the classification of RA (15) were recruited from the Royal Hallamshire Hospital, Sheffield, as previously described; 872 of these patients had erosive damage (5). Research Ethics Committee approval was obtained for the study, and all participants provided informed consent. Radiographs of the hands and feet were obtained at the time of study entry, and modified Larsen scores of radiographic damage progression were obtained as described previously (5,6).

### Measurement of antibodies

Measurement of antibodies was performed as previously described (5). Briefly, anti-CCP antibody levels were measured using the DIASTAT Anti-CCP enzyme-linked immunosorbent assay (Axis-Shield UK, Kimbolton, UK). A cutoff of 5.5 units/ml was established to define antibody positivity, based on the mean (+3 SD) of values obtained for 100 age-matched control subjects. RF was measured using a Dade-Behring BN2 nephelometer (Marburg, Germany).

### Genotyping

Genotyping of SNPs in the IL-1 gene cluster, IL-6, IL-10, PTPN22, SEPS +3705, and SEPS +5227 was performed using TaqMan assays designed by Applied Biosystems (Foster City, CA) (Table 1). Typing of SEPS −105 was performed commercially by personnel at KBiosciences (Herts, UK) using the KASPar method (http://www.kbioscience.co.uk/chemistry/) and primers 5′-GGACGCGGTCGTGGTCCC-3′ (C allele), 5′-GGACGCGGTCGTGGTCCT-3′ (T allele), and common primer 5′-GGTCGGCCTGCGATTGGCC-3′. To ensure high-quality genotyping results, we included multiple positive controls (sequenced templates) and negative controls (ultrapure water templates) in all genotyping plates, 10% of assays were repeated for quality control purposes, and no discrepancies were found. One allelic TaqMan probe was labeled with fluorescent FAM dye and the other with VIC dye (Table 2). The total reaction volume was 5 μl, containing 10 ng of genomic DNA. Thermal cycling in 384-well plates was performed using a PTC-225 DNA Engine Tetrad thermal cycler (MJ Research, San Francisco, CA), and genotypes were determined using an ABI Prism 7900HT Sequence Detection System (Applied Biosystems).

**TABLE 1:** Details of individual single-nucleotide polymorphisms (SNPs)

SNP number	Gene	Description
rs17561	*IL1*α	Ser^114^Ala
rs16944	*IL1*β	Promoter (−511)
rs1143634	*IL1*β	Synonymous coding, Phe^105^Phe
rs419598	*IL1RN*	Synonymous coding, Ala^39^Ala
rs1800872	*IL10*	Promoter (−592)
rs1800896	*IL10*	Promoter (−1082)
rs1800795	*IL6*	Promoter (−174)
rs2476601	*PTPN22*	Arg^620^Trp
Not applicable	*SEPS*	5′-untranslated region
rs4965814	*SEPS*	Intron 5
rs4965373	*SEPS*	3′-untranslated region

**TABLE 2:** Sequences and concentrations of oligonucleotide primers and probes, and annealing temperatures used in polymerase chain reactions for TaqMan-based genotyping*

SNP	Forward primer, 5′→3′	μ*M*	Reverse primer, 5′→3′	μ*M*	Probe, 5′→3′	μ*M*	Temp.
*PTPN22* +1858	CCAGCTTCCTCAACCACAATAAATG	36	CAACTGCTCCAAGGATAGATGATGA	36	VIC–TCAGGTGTCCATACAGG	8	60°C
					FAM–TCAGGTGTCCGTACAGG	8	
*IL1* −511	GTCTCTACCTTGGGTGCTGTTC	36	GAGGCTCCTGCAATTGACAGA	36	VIC–TCTGCCTCAGGAGCT	8	60°C
					FAM–CTGCCTCGGGAGCT	8	
*IL1* +3954	ACCTAAACAACATGTGCTCCACA	36	ATCGTGCACATAAGCCTCGTTA	36	VIC–CATGTGTCGAAGAAGA	8	60°C
					FAM–CATGTGTCAAAGAAGA	8	
*IL1* +4845	TCTGCACTTGTGATCATGGTTTTAGA	36	TGTATTTCACATTGCTCAGGAAGCT	36	VIC–CTAGGTCAGCACCTTT	8	60°C
					FAM–CCTAGGTCATCACCTTT	8	
*IL1RN* +2018	GGGATGTTAACCAGAAGACCTTCTATCT	25	CAACCACTCACCTTCTAAATTGACATT	25	TET–ACAACCAACTAGTTGCCGGATACTTGC	17.5	64°C
					FAM–AACAACCAACTAGTTGCTGGATACTTGCAA	5	
*IL10* −592	GGTAAAGGAGCCTGGAACACATC	36	GCCCTTCCATTTTACTTTCCAGAGA	36	VIC–CCCGCCTGTACTGTAG	85	60°C
					FAM–CCGCCTGTCCTGTAG	8	
*IL10* −1082	GATAGGAGGTCCCTTACTTTCCTCTTA	5	CACACACAAATCCAAGACAACACTAC	30	TET–CCTACTTCCCCTTCCCAAAGAAGCC	15	63°C
					FAM–CTACTTCCCCCTCCCAAAGAAGCCT	5	
*IL6* −174	GACGACCTAAGCTGCACTTTTC	36	GGGCTGATTGGAAACCTTATTAAGATTG	36	VIC–CTTTAGCATGGCAAGAC	8	60°C
					FAM–CTTTAGCATCGCAAGAC	8	
*SEPS* +3705	GCCAGGTTTAGTCTTCTGACACAAA	36	AGCAGGGCCACAGACTTG	36	VIC–CTGACCTTAAACGCTGAGC	8	60°C
					FAM–CTGACCTTAAACACTGAGC	8	
*SEPS* +5227	TTGCCAGACTTATTTTGGGTGAGT	36	TTCAAGGAGATTTATGGACTTCAATTTGTCT	36	VIC–TTTTTATTACAACCTCTAACTATT	8	60°C
					FAM–TTACAACCTCCAACTATT	8	

* SNP = single-nucleotide polymorphism; temp. = temperature.

### Statistical analysis

Hardy-Weinberg equilibrium was examined using the chi-square goodness-of-fit test. Pearson's chi-square test was used to compare allele/genotype carriership between patients and control subjects, and crude odds ratios are presented with 95% confidence intervals. For each DNA variant, the most prevalent homozygous genotype was chosen as the baseline reference.

Dichotomized variables were created for RF and anti-CCP levels (for RF positivity, ≥40 IU/ml; for anti-CCP positivity, ≥5.5 units/ml), and Pearson's chi-square test was used to test for association between allele/genotype and autoantibody seropositivity in patients with RA. Larsen scores of radiographic damage in patients with RA were tested for associations with candidate gene polymorphisms as well as RF and anti-CCP. The Shapiro-Francia test for normality was applied to the data and showed strong evidence against the assumption of normality for the modified Larsen score distribution.

The Kruskal-Wallis nonparametric test was therefore used to compare median scores across genotype groups, followed by the Cuzick nonparametric test for trend to assess gene-dose effects. Genotypes were coded as 0, 1, and 2 for the most prevalent homozygous, heterozygous, and variant homozygous genotypes, respectively. Quantile regression was carried out to estimate the proportion of variance of the different variables in relation to the modified Larsen score. Quantile regression is a complement to classic linear regression, in which the primary goal is to determine the conditional mean of a random variable y, given some explanatory variable x. In quantile regression, one determines the conditional quantile distribution of the y variable; of most common interest is measurement of the 50% quantile or median. All statistical analyses were carried out using Stata statistical software, release 9.1 (Stata Corporation, College Station, TX).

## RESULTS

The study cohort consisted of 964 patients, whose characteristics at baseline are detailed in Table 3. Genotyping data for the SEPS −105 SNP were available for 509 patients; for all other markers, data were available for 856–940 patients in the cohort (89–98%). All genotyping results fit Hardy-Weinberg equilibrium. Because Larsen scores did not fit a normal distribution, we compared median scores across the 3 genotypes. The Shapiro-Francia test for normality was applied to the data and showed strong evidence against the assumption of normality for Larsen score distribution. The Kruskal-Wallis nonparametric test was then applied to test for median Larsen score differences across genotypes of each variant, followed by the Cuzick nonparametric test for trend when a gene-dose effect was observed. Genotypes were coded as 0, 1, and 2 for the most prevalent homozygous, heterozygous, and variant homozygous genotypes, respectively.

**TABLE 3:** Baseline clinical characteristics of the patients with rheumatoid arthritis*

No. of patients	964
Age, mean ± SD years	61.3 ± 12.3
Disease duration, mean ± SD years	15.6 ± 10.7
Female sex, no. (%)	695 (72.1)
Male sex, no. (%)	269 (27.9)
Rheumatoid factor positive, no. (%)	604 (68.4)
Rheumatoid factor negative, no. (%)	279 (31.6)
Anti–cyclic citrullinated peptide positive, no. (%)	697 (76.3)
Anti–cyclic citrullinated peptide negative, no. (%)	217 (23.7)

* Rheumatoid factor status was determined in 883 patients, while antibody status was determined in 914 patients.

The frequency of the IL-6 −174G allele was 55%, and an allele-dose association with increasing radiographic damage was present (*P* = 0.005), with patients with the GG genotype having the highest median modified Larsen score (Table 4). Strong linkage disequilibrium was detected between the 2 IL-10 SNPs (D′ = 0.89 and r^2^ = 0.24), with allele frequencies of 55% and 79% for IL-10 −1082G and −592, respectively. The IL-10 −592CC genotype was associated with greater damage compared with the AC genotype, and the AA genotype was associated with an intermediate score for damage (*P* = 0.006). An allele-dose association of IL-10 −1082 with damage was observed, but the difference in median scores between genotypes (*P* = 0.01) was less than that for IL-10 −592. A relatively weak association of PTPN22 +1858T was detected (*P* = 0.04). The allele frequency of PTPN22 +1858T was 17%, and only a small number of patients had the TT genotype. Radiographic damage increased in an allele-dose manner, with the lowest scores in patients with the PTPN22 +1858CC genotype.

**TABLE 4:** Genotypes and modified Larsen scores of radiographic damage

Gene	Genotype	Patients, no. (%)	Median modified Larsen score*	*P*
*PTPN22* (n = 901)	TT	31 (3.4)	50.0	
	TC	238 (26.4)	33.0	0.04†
	CC	632 (70.1)	25.5	
*IL10* −592 (n = 928)	AA	32 (3.4)	27.5	
	AC	309 (33.3)	22.0	0.006
	CC	587 (63.3)	32.0	
*IL10* −1082 (n = 860)	AA	186 (21.6)	24.0	
	AG	415 (48.3)	27.0	0.01†
	GG	259 (30.1)	31.0	
*IL1RN* (n = 831)	CC	39 (4.7)	36.0	
	TC	334 (40.2)	29.0	0.4†
	TT	458 (55.1)	27.5	
*IL6* −174 (n = 930)	CC	166 (17.8)	25.0	
	CG	482 (51.8)	27.0	0.005†
	GG	282 (30.3)	33.5	
*IL1* −511 (n = 932)	GG	89 (9.5)	33.0	
	GA	421 (45.2)	27.0	0.8
	AA	422 (45.3)	28.0	
*IL1* +3954 (n = 880)	TT	37 (4.2)	49.0	
	TC	332 (37.7)	22.0	0.1
	CC	511 (58.1)	29.0	
*IL1* +4845 (n = 916)	TT	64 (7.0)	31.0	
	GT	398 (43.4)	27.0	0.8
	GG	454 (49.6)	28.0	
*SEPS* −105 (n = 509)	TT	8 (1.6)	49.5	
	CT	128 (25.1)	31.0	0.6
	CC	373 (73.3)	33.0	
*SEPS* +3705 (n = 934)	TT	24 (2.6)	44.5	
	CT	282 (30.2)	28.0	0.2
	CC	628 (67.2)	28.0	
*SEPS* +5227 (n = 888)	GG	99 (11.1)	27.0	
	GA	398 (44.8)	30.5	0.4
	AA	391 (44.0)	26.0	

* Maximum possible score is 160.

† By Cuzick's test for trend.

Dichotomized variables were created for both RF and anti-CCP (for RF positive, ≥40 IU/ml; for anti-CCP positive, ≥5.5 units/ml). Pearson's chi-square test was used to test for association between genotypes and seropositivity. To determine whether the genetic associations with radiographic damage were secondary to the production of RF and anti-CCP antibodies, genotypes were compared between antibody-positive and antibody-negative patients (Table 5). There was a weak association of PTPN22 +1858TT with anti-CCP positivity (*P* = 0.01); however, no other genotypes were significantly associated with antibody production, indicating that the genetic associations were independent of these well-established markers of RA severity.

**TABLE 5:** Genotype frequencies and autoantibody status*

Gene	Genotype	Anti-CCP positive	Anti-CCP negative	OR (95% CI)	*P*	RF positive	RF negative	OR (95% CI)	*P*
*PTPN22* +1858	TT	29	2	5.2 (1.3–45.7)	0.01†	23	5	2.2 (0.8–7.4)	0.1†
	TC	177	50	1.3 (0.9–1.9)		138	76	0.9 (0.6–1.2)	0.3
	CC	446	161			404	190		
*IL10* −592	AA	28	6	1.5 (0.6–4.5)	0.5†	25	8	1.4 (0.6–3.7)	0.6†
	AC	222	72	1.0 (0.7–1.4)	0.9	191	96	0.9 (0.7–1.2)	0.5
	CC	432	138			375	169		
*IL10* −1082	AA	138	36	1.1 (0.7–1.9)	0.6	120	52	1.1 (0.7–1.8)	0.6
	AG	291	113	0.8 (0.5–1.1)	0.2	253	129	1.0 (0.7–1.4)	0.8
	GG	191	57			159	78		
*IL6* −174	CC	120	40	0.9 (0.6–1.5)	0.7	98	56	0.7 (0.5–1.1)	0.1
	CG	351	112	1.0 (0.7–1.4)	0.9	304	140	0.9 (0.6–1.3)	0.6
	GG	207	64			184	77		

* The presence of anti–cyclic citrullinated peptide (anti-CCP) and rheumatoid factor (RF) was compared for each single-nucleotide polymorphism with reference to the commonest genotype. OR = odds ratio; 95% CI = 95% confidence interval.

† By Fisher's exact test for association.

The relationship between antibody status, genotype, and radiographic damage was examined (Table 6). Carriers of IL-10 –592 AC and AA were pooled together because of small numbers. Homozygosity for IL-10 −592C was associated with more severe radiographic damage only in anti-CCP–negative patients (*P* = 0.002) and in RF-negative patients (*P* = 0.002). Conversely, the IL-6 −174G allele was associated with more severe disease only in anti-CCP–positive (*P* = 0.004) patients and RF-positive patients (*P* = 0.01), with an allele-dose effect. Marginally significant associations of PTPN22 +1858T and IL-10 −1082G were also detected.

**TABLE 6:** Modified Larsen scores across genotypes, according to anti-CCP and RF status*

	Anti-CCP positive	Anti-CCP negative	RF positive	RF negative
*PTPN22* +1858				
TT/TC	31.0 (206)	20.0 (50)	42.0 (161)	19.0 (81)
CC	37.5 (445)	8.5 (158)	33.5 (404)	13.0 (188)
*P*	0.5	0.04	0.2	0.07
*IL10* −592				
AA/AC	29.0 (250)	6.0 (76)	31.0 (216)	11.0 (103)
CC	37.0 (431)	16.0 (135)	40.0 (375)	18.5 (168)
*P*	0.06	0.002	0.05	0.002
*IL10* −1082				
AA	27.5 (138)	6.0 (36)	29.5 (120)	8.0 (52)
AG	33.5 (290)	15.5 (110)	37.0 (253)	16.0 (127)
GG	38.0 (191)	10.0 (55)	38.0 (159)	17.5 (78)
*P*	0.02†	0.2	0.1†	0.03†
*IL6* −174				
CC	29.0 (120)	13.0 (39)	30.00 (98)	13.00 (56)
GC	32.0 (350)	13.0 (109)	34.00 (304)	13.00 (139)
GG	41.0 (207)	10.0 (63)	44.00 (184)	10.00 (76)
*P*	0.004†	0.9	0.01†	0.9

* Values are the median Larsen scores (number of carriers). Anti-CCP = anti–cyclic citrullinated peptide; RF = rheumatoid factor; IL-10 = interleukin-10.

† By Cuzick's test for trend.

Finally, the proportion of variance in modified Larsen scores was calculated using the correlation coefficient value (R^2^). As expected in this cross-sectional study, disease duration was the most significant (20%), followed by RF (3%) or anti-CCP status (2%), IL-6 −174 (0.8%), and IL-10 −592 (0.4%).

## DISCUSSION

Established biomarkers for RA severity include RF, anti-CCP, and DRB1 genotypes; however, these explain a relatively small proportion of the variance in disease severity (9). Use of genetic markers predictive of severity has several advantages over use of conventional biomarkers; genotypes are stable, measurable at disease onset, and are amenable to high-throughput assays. Biomarkers can be used to measure inflammation load or bone and cartilage turnover in RA; for example, radiographic damage correlates with the time-averaged C-reactive protein levels (8). New treatments of RA have proven very effective for the treatment of severe disease, but their more widespread use is limited by cost in many countries. Clinically, there is a need to identify additional markers that may be predictive of the outcome of severe disease; such markers could be used to target these new treatments to patients at risk of severe RA. In this study, we examined the association of functional genetic variants in 5 candidate RA genes with radiographic damage, and evaluated whether associations were independent of established biomarkers.

The proinflammatory cytokine IL-6 has a range of pleiotropic activities, including the induction of acute-phase proteins, stimulation of T cells and B cells (including the differentiation and activation of immunoglobulinproducing plasma cells), and the stimulation of synoviocytes and osteoclasts, with resultant damage to cartilage and bone (16). High levels of IL-6 have been reported in RA serum and synovial tissue (17). Furthermore, serum levels of IL-6 have been correlated with the erythrocyte sedimentation rate and the platelet count (18). Our finding of a gene-dose association of IL-6 −174G with radiographic damage may be a consequence of the higher gene expression that has been associated with this allele (19). The IL-6 −174G allele has been associated with susceptibility to systemic-onset juvenile RA in both a large case–control study (19) and a family-based association study (20) but not with susceptibility to RA (21), although it has been associated with a greater inflammation load in RA as assessed by modified Disease Activity Scores as measured in 28 joints (22,23).

IL-10 is produced predominantly by monocytes and lymphocytes and has a range of antiinflammatory and immunoregulatory properties, including inhibition of synthesis of proinflammatory molecules such as TNF, IL-1, and IL-6 (24). Because of these properties, IL-10 was originally named cytokine synthesis inhibitor factor. Production of IL-10 by rheumatoid synovial macrophages and T cells inhibits production of IL-1 and TNF by synovial cells (25), and increased relative expression of IL-10 has been reported in joints without erosion compared with those with erosion, suggesting a protective role in RA (26).

Studies in animal models of RA have also demonstrated an antiinflammatory role of IL-10 (27,28). Genetic factors explain ∼75% of the population variation in IL-10 production (29). Secretion of IL-10 has been correlated with individual promoter SNP genotypes and haplotypes (30,31). Genotypes associated with IL-10 production have been associated with susceptibility to RA (32); however, these findings have not been replicated in other populations (33). Our data are consistent with those from a Dutch prospective study of 91 female patients with RA followed up for 12 years, in whom radiographic damage to the hands and feet progressed more rapidly in individuals homozygous for −1082G compared with those homozygous for −1082A (26). Furthermore, this study also demonstrated lower production of IL-10 in whole blood cultures of individuals with the GG genotype compared with the AA genotype, suggesting that the association of −1082G with radiographic damage is related to lower IL-10 production.

Seropositivity for both RF and anti-CCP is associated with development of more severe erosive RA (5), raising the possibility that our findings of an association of IL-6 and IL-10 genotypes with the modified Larsen score may be secondary to the production of these autoantibodies. However, RF and anti-CCP status was not associated with IL-6 or IL-10 genotypes, indicating that the genetic associations are independent of these markers of radiographic damage.

In our study, the association between the Larsen score and the IL-6 genotype was observed only in patients who were RF negative and/or anti-CCP seropositive (i.e., patients with more severe disease). Conversely, the association between the IL-10 genotype and the Larsen score was present only in patients who were RF negative and/or anti-CCP seronegative (i.e., those with milder disease). These findings may allow insights into the pathogenesis of RA, as well as having potential clinical importance. On the basis of these results, we propose that autoantibody-specific B cells occupy a more upstream position in the processes resulting in the development of RA, and that the influence of particular cytokine polymorphisms on disease phenotype is dependent on their presence or absence. This paradigm is broadly aligned with several recent studies demonstrating the presence of antibodies preceding disease onset (34). From a practical point of view, we suggest that the potential clinical utility of genotyping for the IL-6 and IL-10 polymorphism would depend on RF and anti-CCP status.

The PTPN22 +1858T allele has been associated with susceptibility to autoimmune diseases including systemic lupus erythematosus, type 1 diabetes mellitus, Graves' disease, and RA (4). An area of controversy relates to the association of this variant with RF production. Several groups of investigators reported a genetic association restricted to RF-positive patients 4,35), while other investigators reported that the association is independent of RF status (36,37). We did not observe an association of PTPN22 +1858T with RF status but detected an association of anti-CCP status consistent with that reported by Plenge et al (35).

The cross-sectional design of this study using an RA cohort with established disease did not permit us to examine whether the IL-6 and IL-10 genotypes are associated with a linear increased level of joint damage over time rather than perhaps primarily being important at an early time period; such an assessment will require a prospective study design. Further large prospective studies should clarify the clinical usefulness of genotyping for these SNPs, particularly their potential role in therapeutic targeting of expensive antirheumatic drugs to patients at risk of developing more severe RA.

## AUTHOR CONTRIBUTIONS

Dr. Wilson had full access to all of the data in the study and takes responsibility for the integrity of the data and the accuracy of the data analysis.

**Study design**. Mewar, Moore, Dickson, Binks, Montgomery, Wilson.

**Acquisition of data**. Marinou, Mewar, Moore, Binks.

**Analysis and interpretation of data**. Marinou, Healy, Mewar, Moore, Montgomery, Walters, Wilson.

**Manuscript preparation.** Marinou, Mewar, Moore, Binks, Montgomery, Walters, Wilson.

**Statistical analysis.** Marinou, Healy, Mewar, Walters.
